# On Lancing the Gums

**Published:** 1885-02

**Authors:** Edmund Owen

**Affiliations:** Surgeon to the Hospital for Sick Children, Great Ormond Street


### SELECTIONS.

### On Lancing the Gums.

BY EDMUND OWEN, F.R.C.S., SURGEON TO THE HOSPITAL FOR SICK CHILDREN, GREAT ORMOND STREET.

Read before the Medical Society of London, November 3, 1884.

There are two reasons which, to myself, at least, seem good, for my venturing to bring this short communication before you this evening. The first is, that I have but a slight practical acquaintance with the subject; the second is, that if the question has the real importance which it was formerly considered to have, the sooner one puts himself in the proper course the better. With a due regard to these two objects, I prefer to to throw out suggestions for any discussion that may ensue, rather than attempt to draw conclusions, or advance any theory of my own.

Not many years ago, almost every practitioner carried a silver lancet-case, where now he would carry a thermometer. The lancet then seemed to be used for severing every clinical knot in the maladies of infancy and early childhood which he was not able to untie.

Steadily has the thin-bladed instrument fallen into disuetude. Its place has been filled, I regret to say, by various blades, which, whether used for lancing the gums, for venesection, or for the opening of abscess, are far less suited for the purpose. But to sigh over the sepulchre of the lancet and the leech (for they lie together in the same dark tomb), is not to answer the question as to why the gums are now so rarely lanced. Has an improved condition of gum been evolved as the result of countless years of scarification? Has the process of dentition changed, or have our capabilities in clinical work improved? This last speculation is, I think, worthy of attention; but I am loth to admit it in its entirety, lest one should seem thereby to depreciate the scientific acumen of those many clear-headed and outspoken practitioners who have gone before, and with whom, in their writings, it is often both a pleasure and a profit to dwell.

Dr. Combe, in *The Management of Infancy*, remarks that in the second stage of dentition the gums are very painful and marked with a pale or bright red elevation. He continues, that the infant "snatches at everything, and retains nothing. Nothing pleases him." He then speculates on the shape of the teeth as influencing their eruption, and appears to regard the process of dentition as being, for the most part, a mechanical one. Probably this theory was rather generally held a short time ago: the tooth was on the wrong side of a tough gum, through which the surgeon must help it with his lancet. Even at the present day, the public mind associates dentition with a certain amount of clumsiness or cruelty. How often does one hear such an expression as this: "My children always cut their teeth with diarrhœa, or a large head." Was not the real explanation of all this usually to be found in the fact of the child being improperly fed? The obscure troubles often come on just as the infant is being weaned; and, from what I know on the matter of dieting at about this period, I should be inclined to think that the source of irritation is much more likely to be in the alimentary canal than in the gums. That a slight loss of blood from scarifications of the gums might even in that case give relief I am willing to admit; but I think the saddle must often have been put on the wrong horse. How have the public become possessed of the questionable pathology; and how much truth is there in the theory?

To compare the practice of lancing the infant's gum with that of cutting through a fibrous band or roll of mucous membrane which is preventing the complete emergence of the wisdom-tooth of the adult, is not, I think, quite fair. It has been remarked to me that it is strange that the molar teeth of the child do not demand help from the lancet; and that the sharp-edged teeth, the incisors, are those which are supposed to stand most in need of help. This fact is suggestive.

Dr. Billard remarked that, if all that authors had written on the aberration of the process of dentition should be recorded, an extended chapter of absurdities would be the result. And, quoting Guersant, he says: "Most of the diseases of infancy have been attributed to teething. The difficulty of an accurate observation of diseases at this early age, and the little positive knowledge we possess in this department of pathology, have contributed greatly to the establishment of this opinion." (*Diseases of Infants*, 1839, p. 201.)

There is one special trouble which is apt to be associated with dentition, though not dependant upon it, and which is very apt to be overlooked by the practitioner who is inclined to regard the eruption of the teeth as a morbid process; that is, the early stage of infantile paralysis. I would not pretend that one should be able to foretell the on-coming of the paralysis, when summoned to attend a young child with obscure feverish symptoms; but what I would venture strongly to urge is, that one should not content himself with the suggestion that probably all the symptoms are due to the teething. On several occasions when I have been consulted with regard to the later stages of that disease—the wasted or deformed limbs—I have heard the mother remark reproachfully, if not bitterly, that the doctor assured her that that illness, of many months ago, was but the effect of the teething. I would repeat that dentition is almost invariably a simple physiological process. I would submit that, of the many ills to which tender flesh is heir, few arise from teething, though naturally they accompany it. I would offer a caution, that a careful look-out be kept for one of the most insidious of these evils—essential paralysis.

Then, as regards the gum itself: what is the exact condition which demands scarification? When the alveolus is expanding, the tooth growing, and the enamel advancing, the gum must needs be fibrous and tough; but that it is not really inflamed is evident to the sight, as it is also from the fact that the infant delights in having it pressed and rubbed. Does one often find it red and swollen from inflammation? For years, I have carried a lancet in my card case, but I find no work for it upon the gums. Doubtless there are gums in need of scarification. Who sees them? Who lances them? Do the physicians? What have the family medical attendants to say upon the subject? Is lancing the gums much employed at the present day; is it little employed; is it "a good remedy out of fashion;" or do the rising generation suffer as little from its rather general disregard as they do from the increasing neglect of James' powder?

At the end of the chapter on "Teething," Combe advises that, when there is much local or constitutional disturbance, the gum should be scarified with a lancet, and allowed to bleed freely, though not in the expectation of the tooth immediately following; and that in the second stage, when the tooth is about to appear, the lancing may be demanded for putting an end to suffering and averting danger. I would ask, in conclusion, if this local and constitutional disturbance, resulting from dentition, is often met with now-a-days; and what are the "dangers" that are to be so averted? Last of all, I would like to hear from the dental surgeon as to what might probably have been the effect upon the development of the permanent teeth of the once widely spread practice of lancing the gums.

